# Vascular leak ensues a vigorous proinflammatory cytokine response to Tacaribe arenavirus infection in AG129 mice

**DOI:** 10.1186/1743-422X-10-221

**Published:** 2013-07-02

**Authors:** Eric J Sefing, Min-Hui Wong, Deanna P Larson, Brett L Hurst, Arnaud J Van Wettere, Stewart W Schneller, Brian B Gowen

**Affiliations:** 1 Department of Animal, Dairy, and Veterinary Sciences, Utah State University, Logan, Utah, USA; 2 Institute for Antiviral Research, Utah State University, Logan, Utah, USA; 3 School of Veterinary Medicine, Utah State University, Logan, Utah, USA; 4 Utah Veterinary Diagnostic Laboratory, Utah State University, Logan, Utah, USA; 5 Molette Laboratory for Drug Discovery Research, Chemistry and Biochemistry Department, Auburn University, Auburn, Alabama, USA

**Keywords:** Tacaribe virus, Arenavirus, Animal model, Cytokines, Vascular leak

## Abstract

**Background:**

Tacaribe virus (TCRV) is a less biohazardous relative of the highly pathogenic clade B New World arenaviruses that cause viral hemorrhagic fever syndromes and require handling in maximum containment facilities not readily available to most researchers. AG129 type I and II interferon receptor knockout mice have been shown to be susceptible to TCRV infection, but the pathogenic mechanisms contributing to the lethal disease are unclear.

**Methods:**

To gain insights into the pathogenesis of TCRV infection in AG129 mice, we assessed hematologic and cytokine responses during the course of infection, as well as changes in the permeability of the vascular endothelium. We also treated TCRV-challenged mice with MY-24, a compound that prevents mortality without affecting viral loads during the acute infection, and measured serum and tissue viral titers out to 40 days post-infection to determine whether the virus is ultimately cleared in recovering mice.

**Results:**

We found that the development of viremia and splenomegaly precedes an elevation in white blood cells and the detection of high levels of proinflammatory mediators known to destabilize the endothelial barrier, which likely contributes to the increased vascular permeability and weight loss that was observed several days prior to when the mice generally succumb to TCRV challenge. In surviving mice treated with MY-24, viremia and liver virus titers were not cleared until 2–3 weeks post-infection, after which the mice began to recover lost weight. Remarkably, substantial viral loads were still present in the lung, spleen, brain and kidney tissues at the conclusion of the study.

**Conclusions:**

Our findings suggest that vascular leak may be a contributing factor in the demise of TCRV-infected mice, as histopathologic findings are generally mild to moderate in nature, and as evidenced with MY-24 treatment, animals can survive in the face of high viral loads.

## Introduction

An expanding group of close to 30 viruses comprise the *Arenaviridae* family of viruses. Novel arenaviruses are being discovered, on average, every 2–3 years, and the recent emergence of novel pathogenic arenavirus species suggests that others will be identified in the near future [1,2]. Arenaviruses cause asymptomatic chronic infections in their respective rodent reservoir hosts and are primarily transmitted to humans through inhalation of aerosolized infectious excreta or secretions. Human disease varies widely from asymptomatic or mild febrile illnesses that clear within a few days to severe life-threatening hemorrhagic fever (HF) requiring prompt medical attention. The onset of arenaviral HF generally proceeds inconspicuously with immunosuppression, high viremia, hypercytokinemia, increased vascular permeability, reduced perfusion and eventually hypovolemic shock [3]. There are presently 7 arenaviruses known to cause viral HF. The group includes 5 New World viruses (Junin, Machupo, Guanarito, Sabia, and Chapare) present in areas of South America, and two Old World viruses (Lassa and Lujo) found in defined regions of Western and South Africa [4,5].

The New World arenaviruses (NWA) are comprised of 3 distinct clades (A, B and C) within the Tacaribe complex [6]. Clade B contains the 5 pathogenic arenavirues and several closely related non-pathogenic viruses including Tacaribe virus (TCRV), first isolated in 1956 from a fruit-eating bat (*Artibeuslituratus*) on the island of Trinidad [7]. TCRV is most closely related phylogenetically to Junin virus (JUNV) [4], the etiologic agent of Argentine HF. JUNV has produced the greatest disease burden due to infection by the pathogenic NWA, with case fatality rates ranging from 15-30% in hospitalized patients [8]. Dysregulation of the host innate immune response by JUNV and other pathogenic NWA is believed to impair the development of protective immunity, leading to morbidity and, in many cases, death [3,9].

In addition to public health concerns due to natural transmission of pathogenic NWA in endemic regions of South America, these viruses are considered bioterror agents that could be intentionally released [10]. The lack of safe and effective FDA-approved therapeutic options to treat severe arenavirus infections and the aerosol transmissibility of the viruses contribute to the designation of JUNV and other HF-causing NWA as highest priority category A NIAID pathogens [11], and underscores the need to develop new treatment strategies to counter arenavirus infections. Until recently, mouse models suitable for early stage antiviral drug development had not been described [5]. Lethal JUNV and Machupo virus (MACV) challenge models based on infection of mice deficient in α/β and γ interferon (IFN) receptors [12] or the STAT1 IFN signaling component [13] now provide systems in which promising compounds can be evaluated. However, such studies are generally cost prohibitive due to the requirement of biosafety level (BSL) 4 maximum containment facilities that are not readily accessible to most researchers.

A third mouse model for proof-of-concept antiviral drug studies has also been developed using TCRV, a less biohazardous clade B arenavirus than can be worked with safely in BSL2 laboratories and animal facilities. In addition to describing basic natural history data on the development of viral titers and histopathology during infection, the model was also used to evaluate MY-24, a small molecule that was found to have moderate antiviral activity *in vitro* against TCRV and the Candid vaccine strain of JUNV [14]. Remarkably, the compound was highly effective in preventing mortality in TCRV-challenged mice without reducing viral loads, suggesting that host factors may contribute substantially to the disease pathogenesis. In the present study, we characterize the host response to TCRV infection in the AG129 mouse model and include associated viral titer and histopathology findings to allow direct correlation of the data. We also report on the clearance of virus in surviving mice treated with MY-24, which was not addressed previously.

## Results

**Figure 1:**
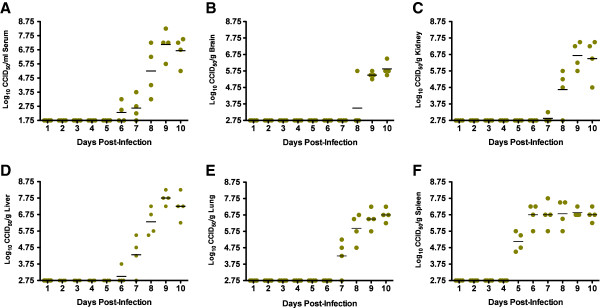
**Temporal analysis of serum and tissue virus titers in AG129 mice challenged with TCRV.** Groups of 4 animals were sacrificed on the specified days of infection for analysis of **A**) serum, **B**) liver, **C**) lung, **D**) spleen, **E**) brain, and **F**) kidney virus titers. One of 5 mice in the day-10 group succumbed prior to the time of sacrifice.

AG129 mice were challenged with a lethal dose of TCRV and groups of animals were sacrificed daily for collection of blood and tissue samples for a comprehensive temporal analysis of the host response during the acute infection. As seen in Figure 1, TCRV was present in the serum and all tissues examined. Virus first became apparent in the circulation on day 6 in 2 of 4 challenged mice, and titers gradually increased until reaching mean levels above 7.25 log_10_ 50% cell culture infectious doses (CCID_50_)/ml on day 9 (Figure 1A). Liver virus was detected in one animal as early as day 6, with peak titers rising to 7.75 log_10_ CCID_50_/g by day 9 (Figure 1B). By gross visual examination, liver samples taken from day 10 mice were a pale mahogany color in 2 of the 4 survivors. Substantial lung virus titers could be detected in 75% of the mice on day 7, with increasing burden thereafter, reaching peak levels (approximately 6.75 log_10_ CCID_50_/g) on day 10 (Figure 1C). Gross examination of lungs revealed necrotic lesions on day 9 and thereafter. The first organ to present with significant amounts of TCRV replication was the spleen, with >4.75 log_10_ CCID_50_/g on day 5 of the infection, and sustained virus burden of >6.5 log_10_ CCID_50_/g out to day 10 (Figure 1D). In several spleen samples collected on and after day 8, gross examination revealed splenomegaly with pale red coloration. TCRV was found in the brain in 1 of 4 animals on day 8 and all infected animals thereafter, with up to 6 log_10_ CCID_50_/g present on days 9 and 10 (Figure 1E). Substantial kidney virus was not observed until day 8 of infection, with the highest mean viral loads of >6.5 log_10_ CCID_50_/g seen on days 9 and 10 (Figure 1F).

**Figure 2:**
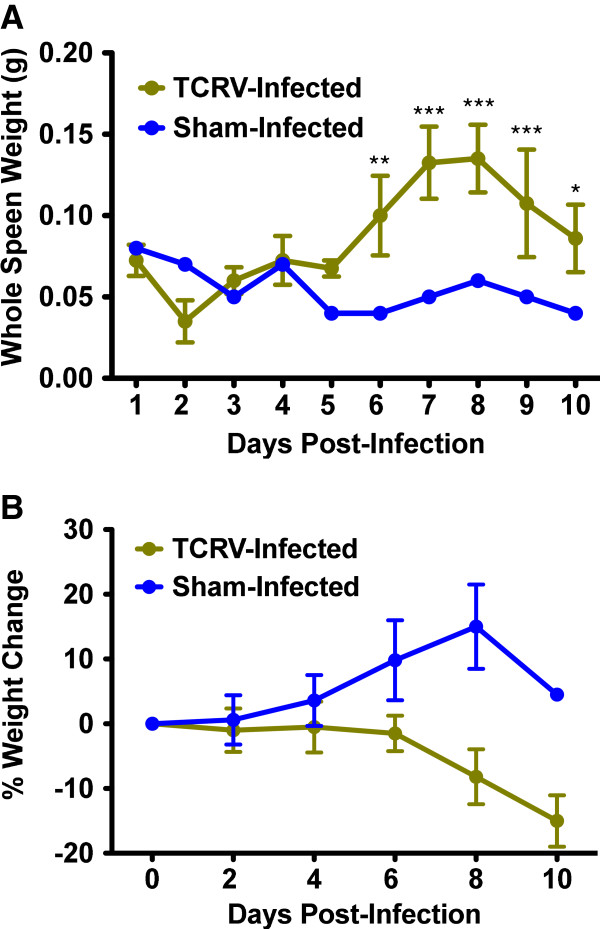
**Whole body weight change and spleen weight in AG129 mice during the course of TCRV infection. A**) Body weight data are represented as the group mean and standard deviation of the percent change in weight of surviving animals relative to their starting weights, measured at 2-day intervals. **B**) Spleen weight data represent the mean and standard deviation from groups of 4 TCRV-infected mice sacrificed at the indicated times. Data from 1 sham-infected control animal per day are included for comparison. n=40, 36, 28, 20, 12, and 4 for the TCRV-infected mice, and n=10, 9, 7, 5, 3, and 1 mouse for the normal controls, on day 0, 2, 4, 6, 8, and 10, respectively. *P< 0.05, **P< 0.01, ****P< 0.001 compared to sham-infected animals.

In addition to the virologic anlaysis, the weights of the mice were measured every other day to limit handling stress. The mean weight of the TCRV-infected mice dropped sharply from day 6 to day 8, with the trend continuing to day 10, the final day of measurement (Figure 2A). The weight loss was consistent with the development of viremia(Figure 1A) and the onset of lethargy and ruffling of fur on day 8 post-infection. Also consistent with the viral titer data, splenomegaly observed in the visual examination was confirmed starting on day 6, with spleens doubling in weight on days 7 and 8 compared to those from the sham-infected controls (Figure 2B).

**Figure 3:**
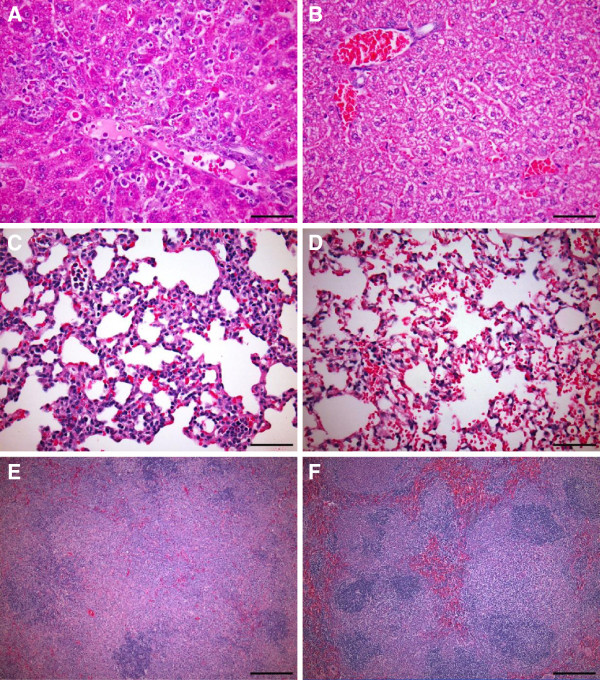
**Histopathology in TCRV-infected AG129 mice at 9 days post-infection.** Representative photomicrographs of liver **A**), lung **C**), and spleen (**E**) from AG129 mice sacrificed on day 9 post-infection with TCRV. Liver **B**), lung **D**), and spleen **F**) from control sham-infected mice. Hematoxylin and eosin staining shows **A**) multifocal moderate histiocytic, neutrophilic and lymphocytic hepatitis (400X), **C**) diffuse moderate interstitial pneumonia (400X), and **E**) white pulp hyperplasia (200X). For panels **A**-**D**, bar = 50 μm; for **E** and **F**, bar = 100 μm.

Histologic examination of livers collected on day 8 and later showed multifocal random hepatocellular necrosis and granulomatous inflammation characterized by infiltration of macrophages, fewer neutrophils and lymphocytes in the hepatic parenchyma (Figure 3A). Lymphocytes, macrophages and fewer neutrophils were also present in the portal tract and multifocally crossed the limiting plate. Glycogen stores in hepatocytes were depleted. Histologic analysis of lungs revealed interstitial pneumonia on day 8 and thereafter (Figure 3C). Examination of spleens on day 8–10 post-infection revealed a moderate to marked increased area and cellularity of the marginal zones due to increased number of atypical mononuclear cells (Figure 3E). There was also a moderate decrease of lymphocyte area and cellularity of the periarteriolar lymphoid sheath and lymphoid follicle as well as decreased red pulp area and cellularity.

**Figure 4:**
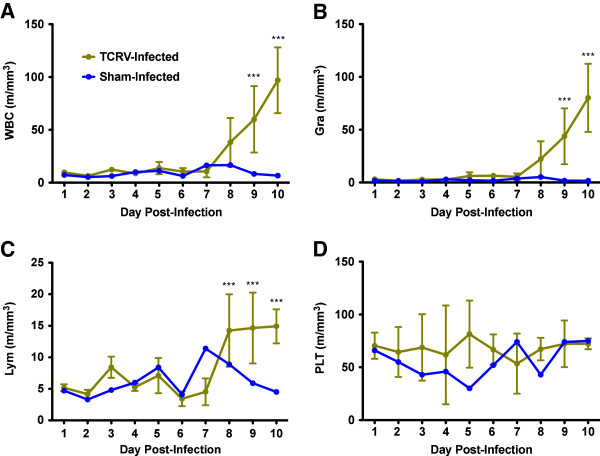
**Hematologic response to TCRV infection in AG129 mice.** Whole blood samples collected from the temporal analysis study were analysed for hematologic parameters (n = 4/day). Notable differences were observed in **A**) white blood cell (WBC), **B**) granulocyte, and **C**) lymphocyte populations, but not **D**) platelet counts. Mean and standard deviations are shown. Data from 1 sham-infected control animal per day are included for comparison.*** *P*< 0.001 compared to sham-infected animals.

Hematologic analysis showed a precipitous increase in white blood cell (WBC) counts starting on day 8, as was also the case for granulocyte and lymphocyte populations (Figure 4A-C). Notably, platelet counts did not decrease in the TCRV-infected animals (Figure 4D), which is a characteristic feature of South American HF [15]. No other significant changes were seen in the hematologic analysis.

Cytokine levels in the mice were also examined from days 5–10 in serum, brain and lung, days 4–10 for kidney, days 3–10 for spleen, and days 3–9 for liver. As shown in Table 1, serum concentrations of a number of cytokines were dramatically increased as the TCRV infection progressed. Proinflammatory mediators such as IL-6, TNF-α, and MCP-1 increased over time during the course of infection. Elevated systemic levels of these and other cytokines have been associated with severe disease in cases of viral hemorrhagic fever [15]. Notably, the lungs had the greatest number of cytokines that were significantly increased or demonstrated a trend towards higher levels as the infection progressed (Table 1). Many of these cytokines were also elevated in liver and spleen tissues. In the brain and kidney, there was very little evidence of an inflammatory response to TCRV. There was a slight trend suggesting increased levels of IL-6, with a more pronounced elevation in MCP-1 in brain samples (Table 1). No significant changes or trends suggesting increased production of cytokines in response to TCRV infection were observed in kidney tissue (data not shown). Collectively, the cytokine profiling data supports a strong proinflammatory response during the evolution of disease in response to TCRV infection in AG129 mice.

**Table 1:** Cytokine response to TCRV infection

						**Days post-infection**				
**Tissue**	**Cytokine**	**Sham-infected**	**3**	**4**	**5**	**6**	**7**	**8**	**9**	**10**
**Serum**	IL-4	9 ± 8	nd	nd	< 4^a^	26 ± 15	38 ± 7	27 ± 7	41 ± 24	44 ± 16
	IL-6	103 ± 75	nd	nd	95 ± 73	516 ± 450	605 ± 192	438 ± 119	1927 ± 1431	3362 ± 3163
	IL-10	12 ± 7	nd	nd	91 ± 22	106 ± 10	111 ± 60	172 ± 29	1110 ± 1055	598 ± 500
	IL-17	10 ± 3	nd	nd	28 ± 1	38 ± 19	34 ± 2	46 ± 20	129 ± 88	68 ± 27*
	MCP-1	81 ± 65	nd	nd	65 ± 8	733 ± 467	749 ± 129	688 ± 255	1257 ± 419**	1590 ± 665***
	TNF-α	35 ± 10	nd	nd	< 7^a^	12 ± 7	57 ± 8	79 ± 32	165 ± 59	244 ± 136*
	MIP-1α	52 ± 49	nd	nd	< 2^a^	24 ± 18	28 ± 22	593 ± 493	1023 ± 613	1041 ± 770
	GM-CSF	30 ± 9	nd	nd	91 ± 22	95 ± 24	178 ± 121	147 ± 81	350 ± 292	371 ± 173
	RANTES	52 ± 17	nd	nd	55 ± 13	89 ± 4	137 ± 56	172 ± 98	402 ± 129**	520 ± 193***
**Brain**	IL-6	306 ± 216	nd	nd	206 ± 8	168 ± 51	446 ± 92	150 ± 102	848 ± 725	516 ± 224
	MCP-1	198 ± 76	nd	nd	90 ± 34	83 ± 1	313 ± 171	211 ± 17	1703 ± 1491	2538 ± 1315
**Liver**	IL-1α	1513 ± 430	1915 ± 575	1841 ± 591	3398 ± 667	3066 ± 390	3293 ± 1234	5436 ± 2061	13500 ± 2089**	nd^c^
	IL-1β	8415 ± 3244	4859 ± 1427	4906 ± 1664	19130 ± 4061	17366 ± 678	15160 ± 4928	16980 ± 4176	54765 ± 8595**	nd^c^
	IL-10	759 ± 239	1457 ± 627	1425 ± 542	1918 ± 329	1569 ± 4	1694 ± 428	1112 ± 387	5010 ± 1110***	nd^c^
	IL-17	728 ± 429	871 ± 275	855 ± 344	1864 ± 383	1486 ± 135	1065 ± 373	1347 ± 681	15409 ± 4366***	nd^c^
	MCP-1	842 ± 298	913 ± 755	955 ± 725	6879 ± 1464**	5426 ± 446*	3326 ± 916	3752 ± 1714	12370 ± 3673***	nd^c^
	GM-CSF	2009 ± 812	2490 ± 976	2494 ± 993	4125 ± 980	3342 ± 131	2556 ± 937	3316 ± 1598	25400 ± 926***	nd^c^
	MIP-1α	2630 ± 867	2464 ± 778	2276 ± 1102	834 ± 60	837 ± 46	3924 ± 1113	12205 ± 10866	80191 ± 17917***	nd^c^
**Lung**	IL-1α	447 ± 134	nd	nd	914 ± 332	518 ± 2	1020 ± 341	632 ± 90	1590 ± 1620	9510 ± 7262*
	IL-2	393 ± 38	nd	nd	490 ± 8	633 ± 15	1690 ± 452	2000 ± 632	3012 ± 1502	8423 ± 7650
	IL-3	392 ± 101	nd	nd	334 ± 36	364 ± 138	355 ± 104	279 ± 45	407 ± 126	2403 ± 101**
	IL-4	401 ± 128	nd	nd	493 ± 286	328 ± 242	786 ± 156	486 ± 94	868 ± 411	3914 ± 2030**
	IL-5	777 ± 110	nd	nd	609 ± 335	590 ± 30	1361 ± 273	891 ± 221	2033 ± 1246	5604 ± 2536***
	IL-6	500 ± 216	nd	nd	276 ± 345	995 ± 314	998 ± 198	1490 ± 1130	2059 ± 815	6680 ± 4214
	IL-10	266 ± 24	nd	nd	< 6^b^	194 ± 235	445 ± 165	1110 ± 942	1620 ± 1511	2340 ± 1174
	IL-12	613 ± 270	nd	nd	129 ± 153	528 ± 568	751 ± 256	485 ± 132	704 ± 218	3348 ± 1584**
	MCP-1	338 ± 74	nd	nd	543 ± 38	1590 ± 245	1506 ± 148	1379 ± 355	3988 ± 3930	23780 ± 23492
	TNF-α	243 ± 200	nd	nd	336 ± 465	186 ± 40	705 ± 341	345 ± 93	1358 ± 1250	8622 ± 4184**
	MIP-1α	565 ± 216	nd	nd	< 8^b^	< 8^b^	1505 ± 148	1379 ± 355	3988 ± 3929	23781 ± 23492
	RANTES	393 ± 38	nd	nd	490 ± 8	633 ± 15	1690 ± 452	2000 ± 632	3012 ± 1502	8423 ± 7650
**Spleen**	IL-1β	5166 ± 2212	20596 ± 1045	35663 ± 16622*	16470 ± 7867	72114 ± 20013***	71460 ± 25186***	17603 ± 4578	17558 ± 7660	15264 ± 15228
	IL-2	302 ± 60	1094 ± 377	1907 ± 871	1428 ± 745	925 ± 413	9384 ± 1767***	2418 ± 1912	471 ± 136	668 ± 296
	IL-6	937 ± 891	315 ± 206	3932 ± 1552	7512 ± 4901	8855 ± 8640	11803 ± 10907	11662 ± 7191	10277 ± 15901	7872 ± 6471
	IL-10	359 ± 181	555 ± 427	2513 ± 1028	9723 ± 6700	2933 ± 3526	34446 ± 12413***	10644 ± 11632	974 ± 475	10010 ± 3066
	MCP-1	65 ± 174	1774 ± 734	3186 ± 1247	2686 ± 1874	10124 ± 2102	8822 ± 6241	10004 ± 6407	11378 ± 8843	37106 ± 34700**
	MIP-1α	2460 ± 1335	3439 ± 457	10489 ± 6500	21290 ± 14825	50613 ± 23941	46596 ± 3376	79600 ± 40143	174281 ± 161060	221110 ± 206073*

^a^ Minimum level of detection for the relevant serum cytokines: < 4 pg/ml for IL-4, < 2 pg/ml for IL-10, < 7 pg/ml for TNF-α, < 2 pg/ml for MIP-1α.

^b^ Minimum level of detection for the relevant lung cytokines: < 6 pg/g for IL-10, < 8 pg/g for MIP-1α.

^c^ Day 10 liver samples were compromised and therefore not included in the analysis.

Not Determined, nd; *P< 0.05, **P< 0.01, ***P< 0.001 compared to sham-infected controls.

**Figure 5:**
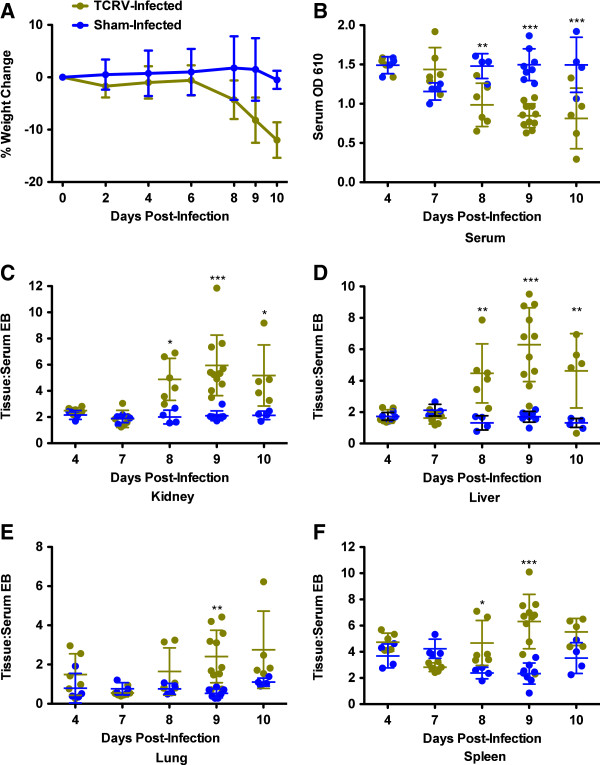
**Evaluation of vascular permeability during TCRV infection in AG129 mice.** TCRV- and sham-infected mice were infused with EBD on day 4, 7, 8, 9 and 10 of infection and systemic levels, as well as leakage into the viscera was evaluated. **A**) Individual mouse weights on day 0, 2, 4, 6, 8, 9, and 10 post-infection were determined and the data are presented as the group mean and standard deviation of the percent change in weight of surviving animals relative to their starting weights. **B**) Serum EBD levels and ratios of tissue to respective serum levels are shown for **C**) kidney, **D**), liver, **E**) lung, and **F**) spleen tissues. Day 9 data include values from two separate experiments. *P< 0.05, ** *P*< 0.01, *** *P*< 0.001 compared to sham-infected animals.

Because it was not possible to measure vascular permeability as part of the initial host response characterization experiment, we conducted a separate time course analysis to track the movement of intravenously injected Evans blue dye (EBD) into various primary tissues in TCRV-challenged mice. As commonly seen, the mice challenged with TCRV exhibited clear signs of clinical illness including lethargy and ruffling of fur by days 8 and 9 post-infection, and the deteriorating condition of the animals was reflected in loss of body weight (Figure 5A). By day 10, the infected mice were approaching a moribund state and the animals that were the most morbid did not show markedly blue extremities following EBD infusion as seen in the sham-infected normal controls (not shown).This is consistent with previously observed lack of perfusion in a model of acute arenavirus infection in hamsters, likely due to hypotension associated with vascular leak [16].

When measuring systemic EBD levels, serum concentrations were reduced in the TCRV-infected control animals on days 8, 9 and 10 post-infection, suggesting leakage of the dye into the viscera (Figure 5B). However, we cannot rule out the possibility that the lower systemic EBD levels seen at advanced stages of illness in the TCRV-infected mice may be due to lack of absorption from the orbital capillary nexus. Therefore, to more accurately access vascular leak of EBD, the tissue concentrations were normalized to amount of dye present in the serum. Liver, kidney, lung and spleen showed a markedly higher mean tissue to serum ratio in the TCRV-infected animals compared to the sham-infected mice on days 8, 9 and 10 (Figure 5C-F), indicating that vascular leakage is occurring and may be an important factor in the demise of the AG129 mice challenged with TCRV.

**Figure 6:**
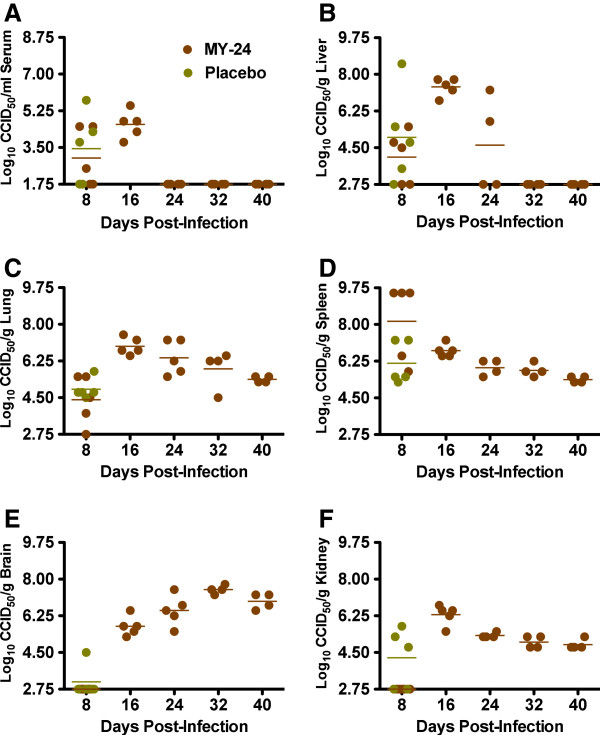
**Analysis of serum and tissue virus titers in AG129 mice infected with TCRV and treated with MY-24.** Groups of 5–6 TCRV-infected animals treated with MY-24 or placebo for up to 8 days starting on day 3 post-challenge were sacrificed on the specified days of infection for analysis of **A**) serum, **B**) liver, **C**) lung, **D**) spleen, **E**) brain, and **F**) kidney virus titers. One mouse per group at days 24 and 32, and 2 mice in the 40-day group succumbed prior to the time of sacrifice and therefore were not included in the analysis.

Prophylactic MY-24 treatment during the first week of TCRV infection has previously been shown to protect mice from mortality in the absence of an appreciable reduction in viral titers, suggesting that components of the host response play a significant role in the pathogenesis of the infection [14]. In the present study, we assessed systemic and tissue viral loads out beyond 5 weeks in surviving animals treated with MY-24 during days 3–10 of the acute infection. Confirming previous observations, MY-24 did not significantly reduce day 8 viral titers in the serum or any tissue compared to mice receiving placebo (Figure 6). Only 3 of the 5 MY-24-treated mice had detectable virus loads in the serum at day 8 post-infection, with all surviving mice having viremia on day 16 (Figure 6A); however, virus was no longer detectable at 24 days post-infection. In the liver, peak viral loads of approximately 7.5 log_10_ CCID_50_/g of tissue were observed on day 16 in the MY-24-treated mice, with virus detected in only 2 of 4 animals on day 24, and absent in all surviving mice at day 32 post-infection (Figure 6B). In the lung, approximately 7 log_10_ CCID_50_/g was detected on day 16, with a gradual decline in titers out to day 40 (Figure 6C). Remarkably, >5 log_10_ CCID_50_/g of lung virus burden was still present at the end of the 40-day study.

**Figure 7:**
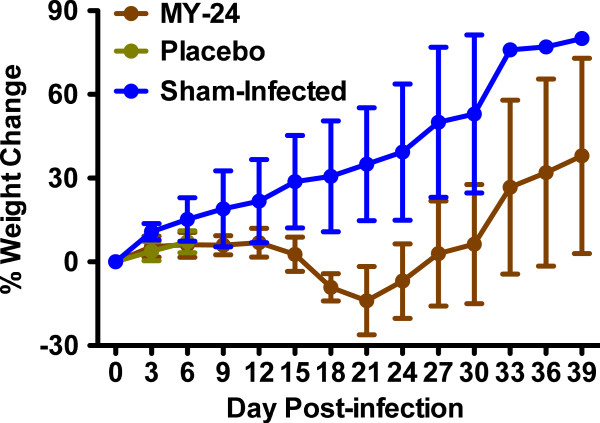
**Analysis of weight change in AG129 mice infected with TCRV and treated with MY-24 or placebo.** Mice from the longitudinal study described in Figure 6 were weighed every third day over the course of the experiment. Normal control mice (n=5, with 1 mouse each sacrificed on days 8, 16, 24, 32, and 40) are included for comparison. The data are represented as the group mean and standard deviation of the percent change in weight of surviving animals relative to their starting weights. Because placebo-treated animals were sacrificed on day 8, only weights through day 6 were determined.

The spleen was the only tissue in which the viral titers trended higher in the MY-24-treated animals on day 8 post-infection (Figure 6D). Notably, 3 of the 5 MY-24-treated mice had ≥9.5 log_10_ CCID_50_/g. Spleen viral burden slightly decreased on days 16–40, but persisted similar to that observed in the lung. On day 8 of the infection, virus was found only in brain tissue from a single placebo-treated animal (Figure 6E). However, substantial titers were observed starting on day 16 ranging from 6 to 7.5 log_10_ CCID_50_/g of brain in surviving MY-24-treated mice. TCRV persistence was also observed in the kidneys of recovering MY-24-treated mice (Figure 6F). As in most tissues, peak titers were observed on day 16 (approximately 6.5 log_10_ CCID_50_/g of kidney), with a subsequent slight reduction in titer as the experiment progressed. In general, MY-24-treated mice began to lose weight after day 12 post-infection and started to recover the lost weight starting on day 21 (Figure 7). These data are consistent with peak titers being observed primarily on day 16, with reductions in virus burden, in most cases, by day 24 of the infection (Figure 6).

**Figure 8:**
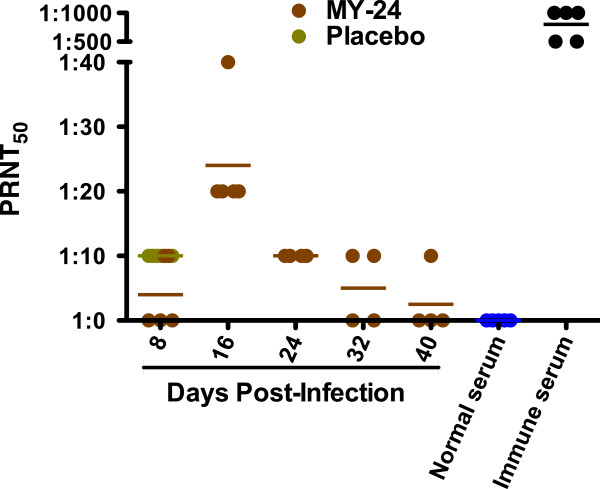
**Neutralizing antibody levels during the course of TCRV infection and recovery in AG129 mice treated with MY-24.** Serum samples from TCRV-infected animals treated with MY-24 or placebo, as indicated in the longitudinal study described in Figure 6, were analyzed for neutralizing antibodies by PRNT. Normal serum from uninfected mice and immune serum were included as controls.

In addition to assessing the viral titers out to 40 days post-infection, we also analyzed neutralizing anti-TCRV titers. At 8 days post-infection, the results suggest that low levels of neutralizing antibodies were elicited earlier in placebo-treated animals (Figure 8). By day 16, PRNT_50_ titers were at their peak in the MY-24-treated mice and waned thereafter. Compared to the PRNT_50_ titers from immune serum from mice that were boosted with live TCRV after having survived infection with a non-lethal challenge dose, the primary neutralizing antibody response was weak to nonexistent in the MY-24-treated mice beyond day 16 (Figure 8). Nevertheless, on day 40, all tissues were histologically normal (data not shown), consistent with the healthy appearance of the mice recovering from the infection.

In a separate experiment, we also assessed vascular permeability in selected tissues from treated mice to assess the impact of MY-24at 9 days post-infection. EBD content in the serum and tissues did not differ significantly between the MY-24- and placebo-treated animals; however, tissue EDB ratios were generally slightly lower in mice treated with MY-24compared to placebo (Additional file 1: Figure S1). Nevertheless, large numbers of mice would have been required to statistically distinguish the subtle, if any, effect that MY-24 may have on limiting vascular leak.

## Discussion

The current paradigm in the viral HF field is that exaggerated release of proinflammatory cytokines into the circulation is an important factor driving the devastating vascular leakage that leads to systemic shock, multi-organ failure, and death [15,17]. Therefore, the treatment of advanced cases of viral HF would likely require a combination of an effective antiviral that directly disrupts the virus life cycle with an agent that can limit the collateral damage caused by an overly aggressive host response. Our data measuring vascular integrity in mice during the acute phase of TCRV infection indicates that vascular leak is a part of the disease process, but its overall contribution to the decline of the animals is difficult to measure. In general, our collective natural history and host response data are consistent with the idea that increasing viral burden in the blood triggers the release of proinflammatory cytokines that drive vascular leak, which likely contributes to the demise of the mice. Indeed, low-level viremia begins on day 6 post infection, at which time elevations in systemic proinflammatory mediators such as IL-6 and MCP-1 are detected, and likely contribute to the increased vascular permeability which begins on day 8, and may contribute to mortality commonly observed between days 10 and 12.Thus, in addition to providing a platform for proof-of-concept studies for early stage antiviral drug development, the TCRV AG129 mouse infection model may also be useful to evaluate strategies under consideration for limiting vascular leak due to sepsis [18].

Previously, we reported on the dramatic protective effect conferred by MY-24 treatment of mice challenged with TCRV [14]. Most impressive was the fact that therapy was still effective even when delaying the initiation of MY-24 treatment as late as 5 days post infection. Remarkably, as confirmed in the present study, treatment did not significantly reduce virus titers, suggesting that the beneficial protective effect may be due to amelioration of the pathogenesis related to an overzealous host response. In a preliminary study investigating the potential effect of MY-24 on vascular permeability at day 9 post-TCRV infection, there was a slight but insignificant reduction observed. This result, combined with limited compound availability, has halted further investigations into the mechanism by whichMY-24 prevents death in AG129 mice infected with TCRV.

In regards to the immune response to TCRV in the AG129 mice, the lack of cytokine production in kidney and brain is consistent with the delay in appearance of virus in those tissues. Furthermore, the subdued proinflammatory cytokine response in kidney and brain tissues likely accounts for the absence of histopathology. The more robust cytokine responses in spleen, liver, and lung tissues were consistent with the more prominent pathology observed in those tissues and suggest that immunopathology plays an important role in the process. Interestingly, the brain was the only tissue in which viral loads increased gradually out to 32 days post infection. This is striking in that systemic virus was cleared by day 24. This suggests that the high and constant titers found in the brains of TCRV-challenged animals on days 24, 32, and 40 were the results of viral replication and persistence in the brain, and not continued seeding from circulating virus.

Arenaviruses are zoonotic agents that produce subclinical infections in their respective rodent reservoir species. An interesting and unexpected finding from our studies was that surviving mice go on to develop chronic infection in various tissues, despite clearing the virus systemically. We found that the weight loss nadir in MY-24-treated mice from days 18 to 21, prior to the recovery of the animals, is consistent with the systemic clearance of the virus between the 16 and 24-day time points. Importantly, however, the AG129 mice lack type I and II IFN receptors, and thus it is difficult to draw conclusions regarding the biological significance of this finding. Arenaviruses likely antagonize native rodent IFN response pathways, while eliciting a controlled cytokine response with minimal disruption of the endothelial barrier. Consequently, one can envisage the evasion of the immune response with limited host-mediated pathogenesis as central mechanisms by which arenaviruses establish chronic carrier states. Additional studies investigating the long-term carriage of TCRV in AG129 mice may lead to new insights in arenavirus-rodent reservoir interactions.

## Methods

### Animals

Six- to seven-week-old AG129 IFN-α/β and –γreceptor-deficient mice were used in these experiments and were obtained from the breeding colony at Utah State University (USU). Mice were age and gender matched for all experiments. They were fed irradiated mouse chow and autoclaved water *ad libitum*. All animal procedures were conducted at the AAALAC-accredited Laboratory Animal Research Center at USU, were approved by the USU Institutional Animal Care and Use Committee (IACUC), and complied with USDA guidelines.

### Virus

TCRV, strain TRVL 11573, was obtained from American Type Culture Collection (ATCC; Manassas, VA). The virus stock (10^6.35^ CCID_50_/ml) used was prepared from clarified liver homogenates from AG129 mice challenged with TCRV (2 passages in Vero 76 African green monkey kidney cells). Virus stock was diluted in sterile minimal essential medium (MEM) supplemented with 50 μg/ml gentamicin and inoculated by intraperitoneal (i.p.) injection.

### Characterization of the AG129 mouse response to TCRV infection

Mice were challenged with approximately 500 CCID_50_ of TCRV and groups of 4 animals were sacrificed daily. Ten sham-infected mice (normal controls) were included in the study for daily sacrifice (one per day) and comparison to the TCRV-challenged animals. Serum and liver, spleen, brain, kidney, and lung tissues were obtained for virus titer determination and cytokine profiling. The spleen from all animals was weighed prior to processing for virus titer. Whole blood collected in EDTA-coated tubes was analyzed for hematology using the VetScan HMT (Abaxis Inc. Union City, CA). A sample of each tissue was preserved in 10% formalin and sent to the Ross A. Smart Veterinary Diagnostic Laboratory (VDL; Logan, UT) for histopathology. Here and elsewhere, clarified tissue homogenates in MEM and serum were stored at −80°C until time of analysis.

### Tissue virus titer determinations

Virus titers were assayed using an infectious cell culture assay as previously described [19]. Briefly, a specific volume of tissue homogenate or serum was serially diluted and added to triplicate wells of Vero 76 cell monolayers in 96-well microtiter plates. The viral cytopathic effect (CPE) was determined 7–8 days post-virus inoculation, and the titers were calculated by endpoint titration [20]. The assay limits of detection were 2.8 log_10_ CCID_50_/g of tissue or 1.8 log_10_ CCID_50_/ml of serum.

### Multiplex cytokine profiling

Tissue and serum concentrations of 16 cytokines(IL-1α, IL-1β, IL-2, IL-3, IL-4, IL-5, IL-6, IL-10, IL-12, IL-17, MCP-1, IFN-γ, TNF-α, MIP-1α, GM-CSF, and RANTES) were evaluated in TCRV-infected mice using Q-Plex mouse cytokine arrays (BioLegend, San Diego, CA) as recommended by the manufacturer. Samples collected on days 2-10 post-infection with TCRV were analyzed for serum and tissue concentrations of the indicated cytokines using the multiplexed array. Samples from normal control animals (n=3-6) were included to establish baseline cytokine concentrations in AG129 mice. The data are presented as pg/ml of serum or pg/g of tissue.

### Assessment of vascular leakage

In a separate time course experiment, vascular permeability was determined by tracking EBD diffusion into various tissues as previously described in a study measuring vascular leak in Pichinde virus and yellow fever virus hamster models of viral HF [16]. Briefly, mice challenged with TCRV were anesthetized at indicated times post-infection with isoflurane and injected retro-orbitally with a 0.5% EBD (Sigma-Aldrich, St. Louis, MO) in phosphate-buffered saline (PBS). Three h after EBD infusion, blood was collected by cheek bleed prior to extensive transcardial perfusion with PBS. Sections of liver, spleen, lung, and kidney tissues were harvested and the dye extracted by overnight incubation in formamide at 37°C. Relative EBD content in the serum was determined from 1:10 diluted samples measured at 610 nm and 740 nm. The absorbance at 740 nm was subtracted from the 610 nm absorbance values to remove contributions due to hemoglobin contamination. Data were expressed as the ratio of absorbance/g of tissue:absorbance of a 10-fold dilution of the respective serum sample.

### Recovery of TCRV-infected mice treated with MY-24

Groups of mice (n=5-6/group) mice were treated i.p. with saline vehicle placebo or 25 mg/kg of MY-24, once daily for up to 8 days starting 3 days post-challenge with 500 CCID_50_ of TCRV. A group of MY-24- and placebo-treated mice were sacrificed on day 8, and the remaining 4-5mice per MY-24 group were sacrificed on days 16, 24, 32 and 40 relative to time of infection. Placebo-treated animals were not included for these later time points because they would not be expected to survive the TCRV challenge. Five sham-infected normal control mice were included in the study for sacrifice at the different time points and comparison to the TCRV-challenged animals. Serum and liver, spleen, brain, kidney, and lung tissues were collected for viral titer analysis. Due to death prior to time of sacrifice, 1 of 5 animals in the 24- and 32-day groups, and 2 of 6 animals in the 40-day group were not included in the analysis.

In a separate experiment using the same challenge dose and treatment regimen, we also determined the effect of MY-24 on vascular permeability on day 9 post-infection. EBD content in the serum and tissues was measured as described above and compared to mice that were treated with placebo, or sham-infected controls.

### Serum plaque reduction and neutralization titers (PRNT)

Neutralizing antibody titers were determined by standard PRNT assay. Briefly, equal volumes of media containing 400 CCID_50_ of TCRV were added to serial 2-fold dilutions of heat-inactivated (56°C for 30 min) serum samples diluted in MEM supplemented with 2% FBS and incubated at 37°C prior to adding the mixtures to 6-well plates seeded with Vero 76 cells. After a 2-h absorption period, the cells were washed with PBS and a 1% sea plaque agarose (Lonza, Rockland, ME) MEM/2% FBS/gentamicin overlay added. Following a day-6 culture period, the cells were stained with neutral red solution and plaques visualized with the aid of a light box. The neutralizing antibody titers were expressed as the highest dilution of serum reducing the average number of plaques present in the virus control wells (TCRV incubated with normal serum) by 50% or greater (PRNT_50_).

### Statistical analysis

For analyzing differences in spleen weights, cytokine levels, hematology parameters, PRNT and vascular permeability, one-way analysis of variance with Newman-Keuls posttests were performed. Day 8 serum and tissue virus titer comparisons between MY-24 and placebo treatments were made using the Mann–Whitney test. All statistical evaluations were done using Prism (GraphPad Software, CA).

## Competing interests

The authors declare no conflict of interest.

## Authors’ contributions

Conceived and designed the experiments: BBG and EJS. Performed the experiments: EJS, MW, and DPL. Analyzed the data: BBG, EJS, DPL and AJVW. Contributed reagents/materials/analysis tools: SWS and BLH. Wrote the paper: EJS and BBG. All authors read and approved the final manuscript.

## Supplementary Material

Additional file 1: Figure S1 Evaluation of vascular permeability in TCRV-infected mice treated with MY-24. TCRV-infected mice treated with MY-24 or placebo, starting 3 days after challenge, were infused with EBD on day 9 of infection and systemic levels, as well as leakage into the viscera, were evaluated. A) Serum EBD levels and ratios of tissue to respective serum levels are shown for B), kidney C) liver, and D) spleen tissue. *P< 0.05, ** *P*< 0.01, *** *P*< 0.001 compared to sham-infected normal animals. Click here for file
