# Supplementary material for: Vascular leak ensues a vigorous proinflammatory cytokine response to Tacaribe arenavirus infection in AG129 mice
Source: Virol J. 2013 Jul 2;10:221. doi: 10.1186/1743-422X-10-221 (PMC3707785; doi:10.1186/1743-422X-10-221)
Supplement: Additional file 1: Figure S1 — Evaluation of vascular permeability in TCRV-infected mice treated with MY-24. TCRV-infected mice treated with MY-24 or placebo, starting 3 days after challenge, were infused with EBD on day 9 of infection and systemic levels, as well as leakage into the viscera, were evaluated. A) Serum EBD levels and ratios of tissue to respective serum levels are shown for B), kidney C) liver, and D) spleen tissue. *P< 0.05, ** P< 0.01, *** P< 0.001 compared to sham-infected normal animals. [file 1743-422X-10-221-S1.docx]

Fig. S1
